# Incidental Thyroid Neuroendocrine Carcinoma Revealing a Presumed Occult Gastrointestinal Malignancy

**DOI:** 10.7759/cureus.117310

**Published:** 2026-10-01

**Authors:** Rebecca J Walker, Fatima Z Aly, Matthew Gorris, Allison Slijepcevic, Christopher A Sullivan

**Affiliations:** 1 Department of Otolaryngology-Head and Neck Surgery, Atrium Health Wake Forest Baptist, Winston-Salem, USA; 2 Department of Pathology, Wake Forest School of Medicine, Winston-Salem, USA; 3 Department of Endocrinology, Atrium Health Wake Forest Baptist, Winston-Salem, USA

**Keywords:** dotatate pet, gastrointestinal neuroendocrine tumor, incidental thyroid mass, metastatic neuroendocrine tumor, neuroendocrine carcinoma, octreotide, somatostatin receptor-positive tumor, thyroid metastasis

## Abstract

In rare cases, a thyroid mass may represent metastatic neuroendocrine disease rather than a primary thyroid malignancy. This case report describes a unique presentation of neuroendocrine carcinoma involving the thyroid with concurrent somatostatin receptor-positive gastrointestinal and mesenteric lesions of uncertain origin, providing insight into the diagnostic and management challenges of this disease. We present a 60-year-old male patient found to have an incidental 11 cm left thyroid mass with airway displacement on neck computed tomography (CT). A left thyroid lobectomy was performed, and surgical pathology confirmed neuroendocrine carcinoma. Immunohistochemical and molecular testing argued against medullary thyroid carcinoma, while site-of-origin markers created a diagnostic challenge with features suggestive of both pulmonary and non-pulmonary origin. Subsequent DOTATATE positron emission tomography (PET) scan demonstrated somatostatin receptor-avid lesions involving the duodenum and mesentery, raising the possibility of a gastrointestinal primary with metastatic spread to the thyroid. The discordance between the immunohistochemical profile and imaging findings highlights the diagnostic complexity of neuroendocrine tumors involving the thyroid and underscores the importance of comprehensive systemic evaluation, including functional imaging and site-of-origin immunohistochemistry, in the setting of atypical thyroid pathology. Prompt recognition of potential systemic neuroendocrine malignancy is essential for staging and therapeutic planning.

## Introduction

Neuroendocrine carcinomas are rare neoplasms that can metastasize to various organs, though thyroid involvement is exceedingly uncommon [1]. Metastatic lesions to the thyroid are rare and often clinically silent [2-5]. When neuroendocrine tumors are found simultaneously in other organs as well as the thyroid, it can be difficult to determine the initial site of malignancy and the extent of disease [6,7]. Somatostatin receptor expression varies across neuroendocrine neoplasms but can provide an important target for functional imaging and, in selected tumors, somatostatin analog therapy, making imaging with radiolabeled somatostatin analogs a mainstay in the evaluation of overall disease burden and somatostatin analogs such as octreotide a useful tool to slow tumor progression [1,8-10]. Classification of neuroendocrine tumors is broad, and treatment options can vary, though a combination of surgical resection and medical management is often warranted [1,8]. Pathologic evaluation of suspected tumor sites is essential to identify the origin of the disease and appropriately grade neuroendocrine tumors to provide appropriate treatment recommendations.

## Case presentation

A 60-year-old male patient with a past medical history of hypertension, lymphedema, hyperlipidemia, and chronic obstructive pulmonary disease (COPD), as well as former smokeless tobacco use, was found to have a 3 cm squamous cell carcinoma of the right lower lip. He underwent a neck computed tomography (CT) that showed an incidental 11 cm left thyroid mass with associated airway displacement, a large periparotid 5 cm mass, a 1 cm level 1b lymph node, and bilateral internal jugular vein lymph nodes (Figure 1).

**Figure 1:**
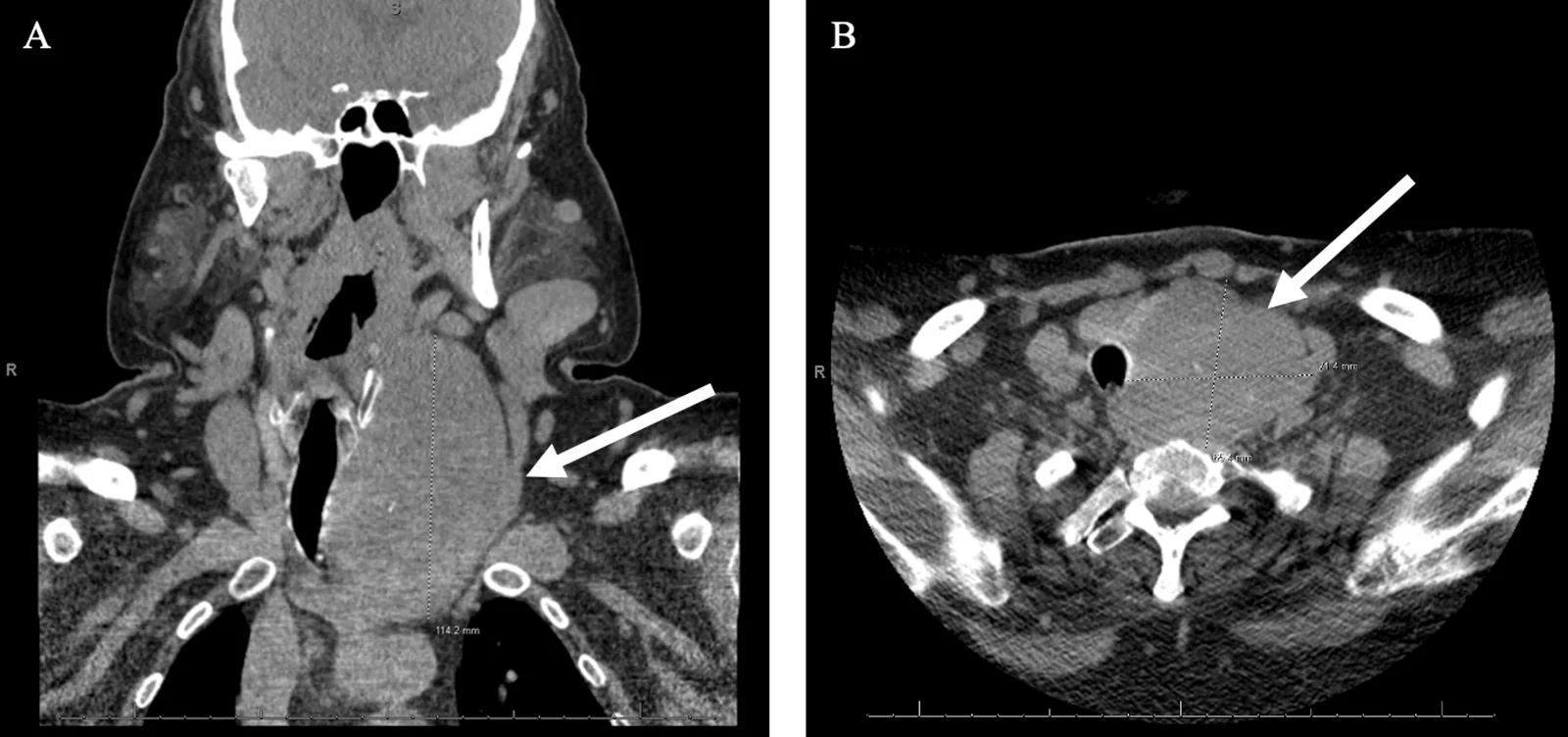
CT imaging of the neck demonstrating a thyroid mass. (a) Coronal CT image demonstrating a large left-sided thyroid mass (arrow) measuring approximately 11 cm with significant rightward airway displacement. (b) Axial CT image showing the extent of the left thyroid mass (arrow) and associated tracheal deviation. CT: computed tomography

A left thyroid lobectomy was performed, and level 1a and 1b lymph nodes were resected. Initial surgical pathology of the thyroid specimen showed evidence of infiltrating tumor composed of large cells with prominent nucleoli, concerning for neuroendocrine carcinoma with differentials including medullary thyroid carcinoma and large cell neuroendocrine carcinoma of the lung. The tumor was confined to the thyroid gland and was hemorrhagic without evidence of necrosis. Lymph nodes were negative for metastatic carcinoma. Rare mitoses were seen (3 per 2 mm²), and the Ki-67 proliferation index, assessed by manual counting of 500 cells, was up to 20%. Immunohistochemical staining of the thyroid specimen was positive for synaptophysin (diffuse) and chromogranin A (patchy), confirming neuroendocrine differentiation. Insulinoma-associated protein 1 (INSM1) was negative (Figure 2). Cytokeratin 7 (CK7) stain demonstrated patchy positivity. Thyroid transcription factor 1 (TTF-1) was also positive, raising the consideration of pulmonary origin; however, subsequent cross-sectional imaging demonstrated no pulmonary mass. Stains for medullary thyroid cancer (calcitonin and carcinoembryonic antigen (CEA)) were negative, and thyroglobulin was negative. Serum calcitonin, CEA, and chromogranin A were normal within the reference range (Table 1). Next-generation sequencing studies demonstrated a lack of RET mutation or translocation.

**Figure 2:**
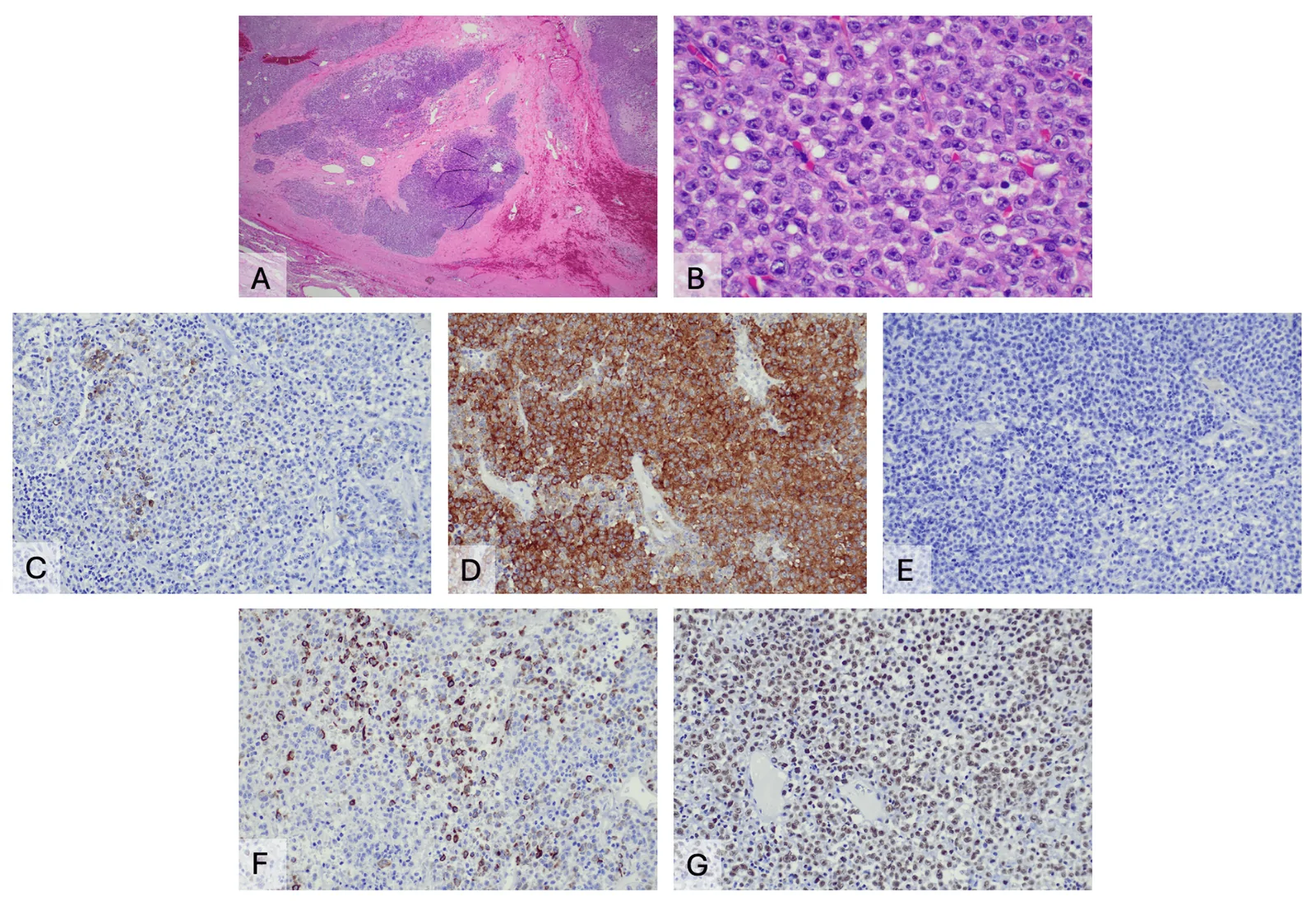
Histopathology images of the thyroid tumor and immunohistochemical stains. (A) H&E-stained section (×20). (B) H&E section (×200) showing a mitotic figure. Immunohistochemical stains at ×200 magnification showing patchy positive staining for chromogranin A (C) and diffuse positivity for synaptophysin (D) suggesting a neuroendocrine carcinoma phenotype. Additional stains are negative for INSM1 (E), with patchy positivity for CK7 (F) and positivity for TTF-1 (G). H&E: hematoxylin and eosin; INSM1: insulinoma-associated protein-1; CK7: cytokeratin 7; TTF-1: thyroid transcription factor 1

**Table 1:** Serum tumor markers. CEA: carcinoembryonic antigen

Serum labs	Value	Reference values
			Calcitonin	4.1	0.0-8.4 pg/mL
CEA	1.1	<3.0 ng/mL
Chromogranin A	99.3	0.0-101.8 ng/mL

To assess a follicular cell origin, the monoclonal antibody PAX8 (paired box gene 8; clone BC12, BioCare, Pacheco, CA, USA), a marker expressed in thyroid follicular cell neoplasms, was applied and was negative. To screen for alternative primaries and mimics, additional stains were negative for neurofilament protein (NFP), alpha-methylacyl-CoA racemase (AMACR), paired box gene 5 (PAX5), special AT-rich sequence-binding protein 2 (SATB2), and NK3 homeobox 1 (NKX3.1). Cross-sectional imaging showed no pulmonary mass.

Post-operative imaging with DOTATATE positron emission tomography (PET) scan demonstrated previously undiagnosed somatostatin receptor-positive lesions involving the duodenum and mesentery, raising concern for additional disease (Figure 3).

**Figure 3:**
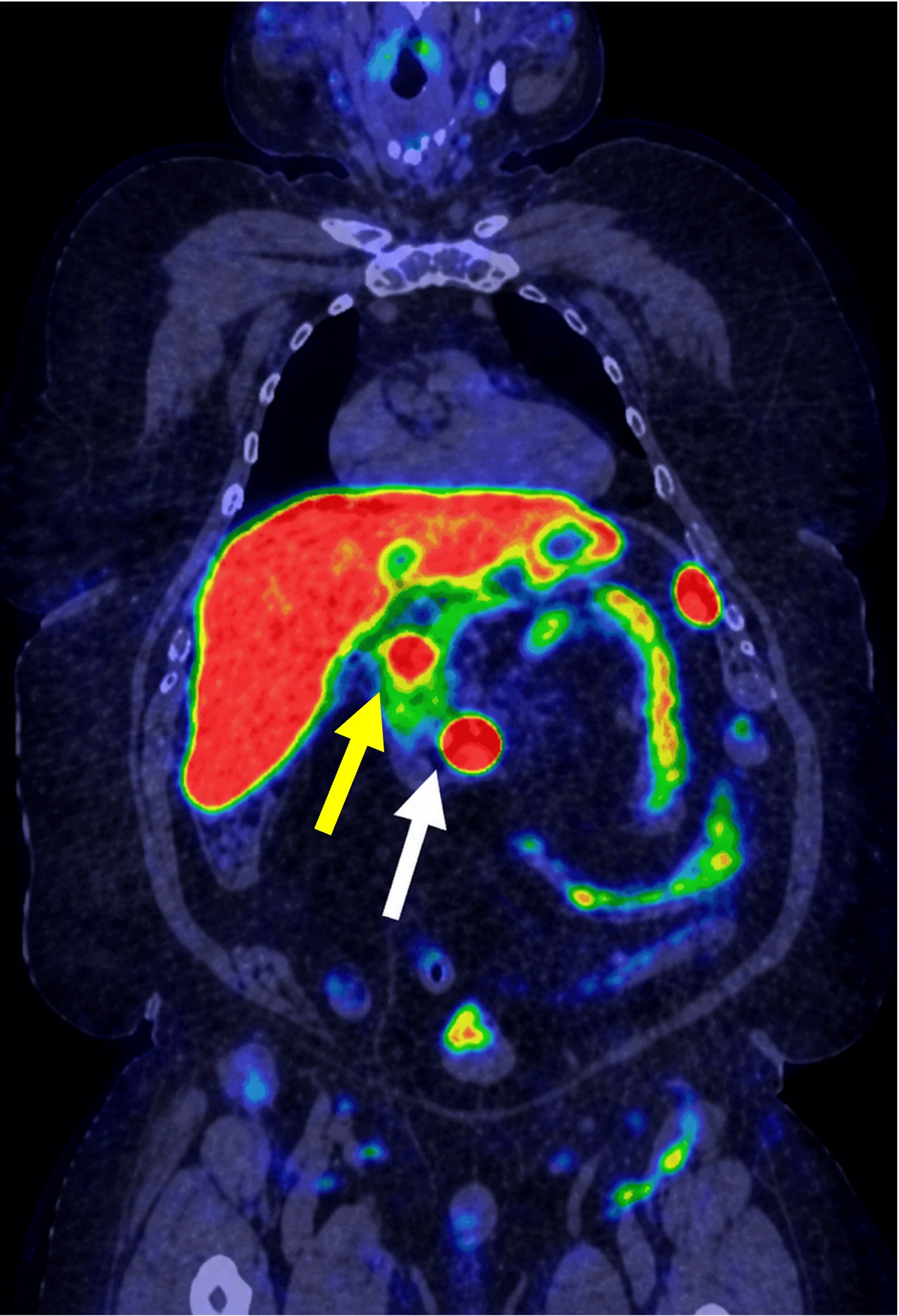
DOTATATE PET imaging demonstrating a somatostatin receptor-positive disease. PET imaging revealing intense radiotracer uptake in the duodenum (yellow arrow) and mesentery (white arrow), findings that are suspicious for neuroendocrine tumor involvement of the gastrointestinal tract. PET: positron emission tomography

The primary site remained uncertain; however, the distribution of disease raised suspicion for a possible primary gastrointestinal neuroendocrine neoplasm of the duodenum with metastatic involvement of the thyroid. Abdominal magnetic resonance imaging (MRI) also demonstrated paraduodenal and mesenteric soft tissue masses in the upper abdomen, similar to lesions identified on DOTATATE PET scan. In addition, the abdominal MRI revealed bilateral iliac chain lymphadenopathy.

Further recommendations in the patient's care included endoscopic evaluation with biopsy of the duodenal lesion and resection of duodenal and mesenteric lesions. Given the lack of evidence pointing to medullary thyroid carcinoma, further surgical intervention with completion thyroidectomy and neck dissections was deferred. Histologic confirmation of a gastrointestinal primary could not be obtained because the patient declined further biopsy or resection of the duodenal and mesenteric lesions. The patient was only willing to undergo empiric long-acting octreotide therapy, and octreotide LAR 30 mg was administered intramuscularly monthly to slow tumor growth and progression (Figure 4).

**Figure 4:**
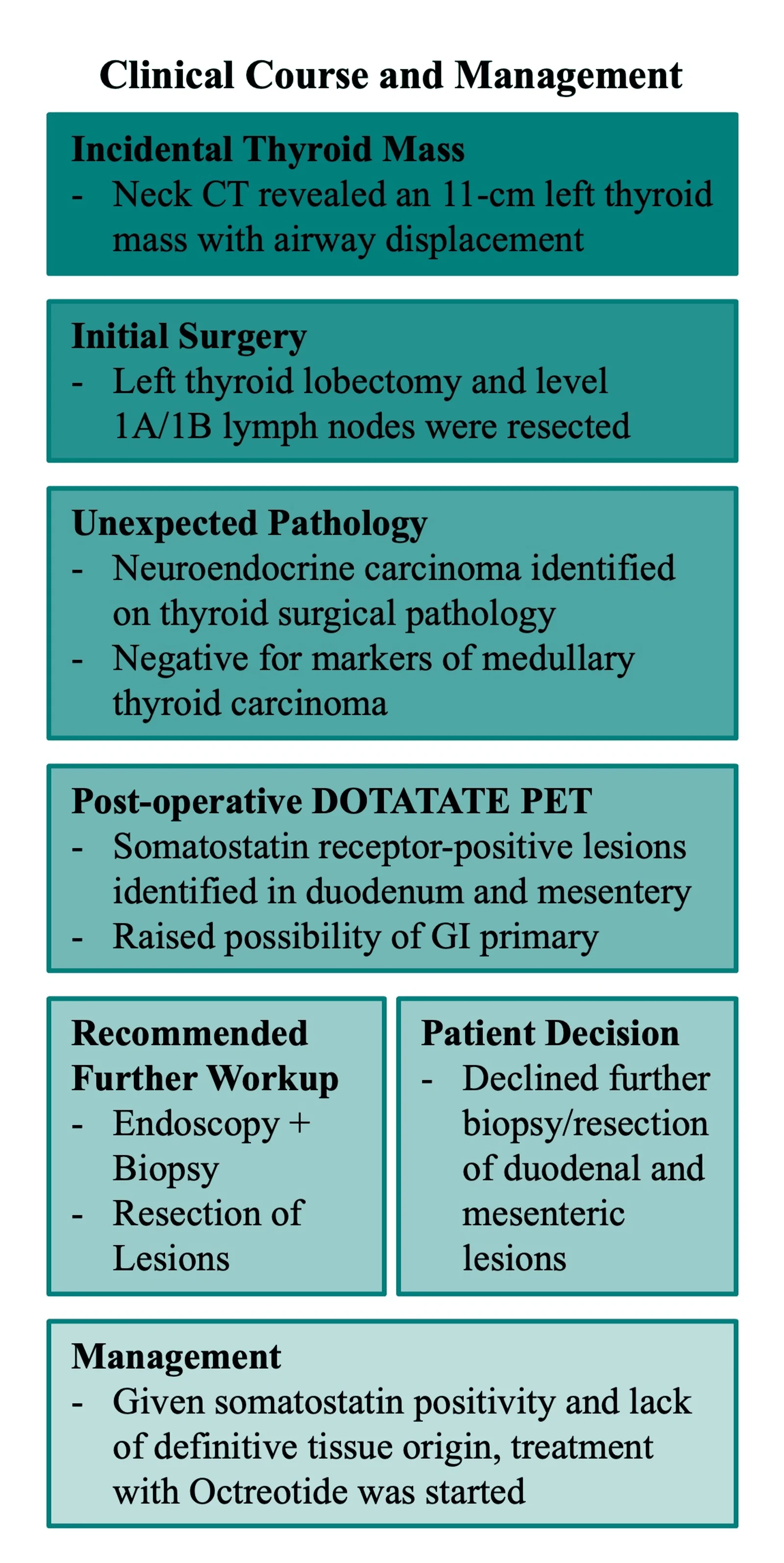
Clinical course and management of neuroendocrine carcinoma of unknown origin found in the thyroid. PET: positron emission tomography; GI: gastrointestinal; CT: computed tomography

## Discussion

Our case study describes a unique presentation of neuroendocrine carcinoma involving the thyroid with concurrent somatostatin receptor-positive gastrointestinal and mesenteric lesions of uncertain origin, providing insight into the diagnostic and management challenges of neuroendocrine carcinoma. Diagnosis and workup of neuroendocrine carcinoma can be extensive [1]. The most common neuroendocrine tumor of the thyroid is medullary thyroid carcinoma; however, negative testing for calcitonin (both immunohistochemical and serum), CEA, thyroglobulin, and RET mutation strongly argues against the diagnosis in this case [1]. In atypical cases where a neuroendocrine carcinoma involves the thyroid and is not consistent with medullary thyroid carcinoma, there should be a high index of suspicion for both primary malignancy in other areas of the body and metastatic disease [5,7].

Metastasis to the thyroid is rare, with the hypothesis being that high iodine content with rapid arterial flow creates a unique microenvironment that makes the thyroid a less favorable location for tumor implantation [3]. Among neuroendocrine tumors specifically metastasizing to the thyroid, thoracic primaries (lung and thymus) are the most commonly reported [6]. The immunohistochemical profile of this tumor presents a diagnostic challenge. TTF-1 positivity with concurrent PAX8 and thyroglobulin negativity can be indicative of pulmonary origin, as TTF-1 is positive in approximately 50-95% of pulmonary carcinoids and is characteristically negative in gastrointestinal and pancreatic neuroendocrine tumors [11-13]. However, in this case, there was no evidence of a pulmonary mass. Thyroid follicular cell-derived neoplasms characteristically co-express TTF-1 and PAX8, so the absence of PAX8 argues against a follicular cell-derived thyroid neoplasm [14]. It does not, however, exclude a primary thyroid neuroendocrine (C-cell-derived) tumor, as medullary thyroid carcinoma is commonly PAX8-negative. In this case, the negative calcitonin, CEA, and RET results instead argue against a conventional medullary carcinoma, leaving a calcitonin-negative neuroendocrine tumor as the working diagnosis.

Due to the large size of the thyroid mass and impending airway compromise in this patient, there was a strong indication for initial thyroid lobectomy. The tumor demonstrated somatostatin receptor expression, and post-operative DOTATATE PET imaging identified somatostatin receptor-positive lesions in the duodenum and mesentery, raising concern for additional neuroendocrine disease. Given this finding, the possibility of a primary gastrointestinal neuroendocrine tumor with metastasis to the thyroid was raised. However, these lesions were not biopsied because the patient declined further invasive evaluation, and therefore, a gastrointestinal primary tumor and metastatic spread to the thyroid could not be histologically confirmed. The discordance between the thyroid tumor's immunohistochemical profile and the distribution of somatostatin receptor-positive disease further complicated the determination of the primary site. Additional tissue sampling of the gastrointestinal lesions would be necessary to establish the primary site of origin and tumor biology, as well as distinguish well-differentiated from poorly differentiated neuroendocrine carcinoma [1].

Tumor classification and primary site identification have important implications for the treatment of neuroendocrine neoplasms. Somatostatin analogs have demonstrated antiproliferative benefit primarily in well-differentiated grade 1-2 neuroendocrine tumors, as established in the PROMID and CLARINET trials [9,10]. A site-specific tumor grade was not assigned because the thyroid lesion was classified as neuroendocrine carcinoma rather than medullary thyroid carcinoma and the primary site remained unknown. The suspected gastrointestinal lesions were not biopsied, preventing histologic confirmation and site-specific classification of a potential gastrointestinal primary. The lack of further classification limited treatment recommendations, and the patient was treated empirically with octreotide due to the somatostatin receptor positivity of the lesions. Although the NETTER-2 trial demonstrated benefit of first-line lutetium Lu 177 Dotatate plus octreotide in patients with advanced gastroenteropancreatic neuroendocrine tumors and Ki-67 ≥10%, the applicability of these findings to this patient is uncertain because the primary site and site-specific tumor classification were not established [15]. If further evaluation of the duodenal and mesenteric lesions was obtained, it would be essential to determine the Ki-67 proliferative index, which could help establish a grade and guide specific management.

Metastasis of gastrointestinal neuroendocrine tumors to the thyroid has been reported only in a limited number of cases [6]. Prognosis of metastatic somatostatin receptor-positive gastrointestinal neuroendocrine tumors is highly variable and dependent on the primary site of malignancy, tumor grade, and differentiation. For distant-stage small intestine neuroendocrine neoplasms, a recent Surveillance, Epidemiology, and End Results (SEER) analysis reported a median overall survival of 8.2 years, though outcomes are substantially worse for poorly differentiated neuroendocrine carcinomas (median overall survival of 35.7 months for small intestine neuroendocrine carcinoma) [16]. In this case, somatostatin receptor positivity provided a rationale for empiric treatment with long-acting octreotide, although the expected benefit of somatostatin analog therapy remains uncertain given the unresolved primary site and tumor classification. These treatments can help slow tumor progression; however, long-term multifocal metastatic disease is difficult to control.

## Conclusions

Incidental detection of a neuroendocrine neoplasm involving the thyroid can serve as the initial clue to an underlying, systemic neuroendocrine malignancy. A calcitonin-negative neuroendocrine neoplasm involving the thyroid should prompt investigation for a possible non-thyroid primary tumor. The immunohistochemical profile is important for evaluating possible site of origin but should be interpreted alongside tumor morphology, clinical findings, and systemic imaging. Prompt recognition and functional imaging are essential for staging and therapeutic planning for atypical neuroendocrine carcinoma presentations. A multidisciplinary approach involving surgical, endocrine, and oncology teams is critical for optimizing evaluation and management of patients with possible metastatic neuroendocrine disease.
